# Down the Digital Delta: Health Information Inequities Among Rural Mississippi Caregivers

**DOI:** 10.3390/healthcare13182361

**Published:** 2025-09-19

**Authors:** Danielle K. Nadorff, Sujan Anreddy, Katerina Sergi, Zaccheus J. Ahonle, Colleen Stouffer, Tockie Hemphill, David R. Buys

**Affiliations:** Social Science Research Center, Mississippi State University, Starkville, MS 39762, USA; sujan.anreddy@ssrc.msstate.edu (S.A.); ksergi@ssrc.msstate.edu (K.S.); zja34@msstate.edu (Z.J.A.); colleen.stouffer@ssrc.msstate.edu (C.S.); tockie.hemphill@ssrc.msstate.edu (T.H.); david.buys@msstate.edu (D.R.B.)

**Keywords:** digital health literacy, health information seeking, rural caregivers, Digital Divide Theory, Theory of Planned Behavior

## Abstract

**Background/Objectives**: As healthcare increasingly utilizes digital delivery systems, equitable access and engagement are critical, particularly for caregivers of older adults in rural regions. This study examines how education levels and geographic rurality influence health information-seeking in Mississippi, a state with persistent structural inequities, through the theoretical lenses of Digital Divide Theory and Theory of Planned Behavior. **Methods**: A statewide survey was conducted among caregivers in Mississippi (N = 452) who support adults aged 50+. The survey assessed rurality level, educational attainment, attitudes toward various health information sources, perceived digital accessibility, and reported challenges in obtaining necessary health guidance. **Results**: Findings challenged conventional assumptions regarding rural digital engagement. Rural caregivers reported higher trust in both internet and interpersonal health information sources. Rurality did not significantly predict internet use or reported difficulty finding information. However, a significant interaction between education and rurality revealed an “Outcome Divide”: while higher education correlated with more positive attitudes toward online health information in urban areas, this association weakened and reversed in highly rural contexts. **Conclusions**: These results underscore the need for strategies beyond merely improving access to bridge digital health equity gaps. Policy and interventions must address contextual barriers, such as digital health literacy and relevance, limiting the effectiveness of digital tools, even when internet access is available. Promoting digital health literacy, integrating trusted local interpersonal networks, and adapting educational initiatives to rural realities are essential for advancing equitable and effective digital health engagement.

## 1. Introduction

### 1.1. Background and Public Health Context

More than 53 million people in the United States serve as unpaid caregivers, with approximately 41 million caring for an older adult [1]. These caregivers provide essential support ranging from assistance with daily living and medical tasks to emotional and financial support, often without formal training or compensation. Their efforts are vital to sustaining the health and independence of older adults, particularly in the face of increasing chronic disease prevalence and long-term care needs. However, the challenges faced by caregivers are not uniform across the country. Rural caregivers, who comprise a significant share of this caregiving population, often encounter distinct obstacles due to their geographic location. They are disproportionately affected by healthcare workforce shortages, limited respite care, and long travel distances to access essential health services [2]. This physical isolation is often compounded by digital exclusion, as broadband access in rural communities remains significantly lower than in urban areas, limiting caregivers’ ability to access telehealth services, educational resources, or virtual support groups [3]. Furthermore, rural caregivers are more likely to report emotional stress and lack of access to formal caregiving support networks, increasing their risk of caregiver burnout [4].

These significant challenges underscore why access to reliable, understandable, and timely health information is vital for caregivers to effectively support the well-being of older adults and other care recipients [5]. Caregivers are often responsible for complex medical tasks, such as managing medications, scheduling appointments, monitoring symptoms, and navigating health insurance, frequently despite limited formal training [1]. Without credible information, caregivers risk making decisions based on misinformation, which can lead to medication errors, delayed treatment, and increased hospitalizations. Conversely, reliable health information empowers caregivers to make informed decisions about care plans and treatment options, communicate effectively with healthcare providers, recognize early warning signs of health deterioration, understand chronic conditions, mental health challenges, and mobility issues, and connect with community resources such as respite care, telehealth, or home-based services.

This need for trustworthy health information is especially urgent in rural or underserved communities, where caregivers may face additional barriers such as poor internet access or low digital literacy. In such contexts, misinformation can spread more easily, and the absence of accessible resources increases caregiver burden and stress. Therefore, improving caregivers’ access to reliable health information, through strategies like user-friendly platforms, culturally appropriate materials, and digital literacy training, holds significant potential to improve care quality, reduce disparities, and enhance both caregiver and recipient outcomes [6].

These national trends and rural challenges are particularly applicable in Mississippi, one of the most rural states in the U.S. As of 2025, approximately 54.4% of Mississippi’s population resides in rural areas, compared to the national average of about 19% [7], and the state is home to roughly 470,000 caregivers [8]. This predominantly rural distribution contributes to unique and persistent challenges in healthcare access, digital infrastructure, and socioeconomic outcomes, particularly for older adults and their caregivers. Mississippi faces severe provider shortages, with 76 of its 82 counties designated as Health Professional Shortage Areas (HPSAs) for primary care, mental health, or dental services [9]. Rural hospitals across the state have closed or downsized due to financial instability, forcing residents to travel long distances for even basic medical care. Compounding this, rural Mississippians are more likely to be uninsured or underinsured, limiting access to preventive and chronic care services. The state also ranks among the lowest in broadband availability and adoption. According to the FCC [10] and Mississippi Broadband Office (BEAM) [11], over 40% of rural Mississippians lack access to reliable high-speed internet, creating a digital divide that impedes telehealth use, online health information access, education, and employment opportunities. This gap is particularly harmful for caregivers and older adults who increasingly depend on digital tools for health management, communication, and support services. Furthermore, high poverty rates, low educational attainment, transportation barriers, and racial inequities further exacerbate health disparities across rural Mississippi. These structural factors collectively hinder efforts to achieve health equity and digital inclusion, necessitating targeted policies and community-driven solutions. To examine these dynamics, this study operationalizes ‘rurality’ using the U.S. Department of Agriculture’s 9-point Rural-Urban Continuum Codes (RUCC), which classify counties along a spectrum from major metropolitan centers to completely rural areas [12].

### 1.2. Technology as a Tool for Health Equity

Amidst these challenges, technology, particularly the internet, has emerged as a potentially powerful tool. Over the past two decades, the internet has rapidly become one of the most important sources of health information for the public [13]. According to the Pew Research Center, nearly eight in ten U.S. adults have searched for health information online, seeking information on specific diseases, treatments, medications, and preventive care strategies [14]. This shift has been fueled by factors such as increased digital access, growing trust in reputable online resources, and the expansion of telehealth. With smartphones and broadband becoming more widespread (albeit unevenly distributed), health information is virtually available anytime and anywhere for those connected. Major health organizations have strengthened their online presence, providing up-to-date, evidence-based guidance, and popular platforms like WebMD, PatientsLikeMe, and health forums offer accessible, immediate information that can empower individuals to take an active role in their healthcare.

However, this reliance on online resources is not without risks and challenges. The quality and credibility of online information vary widely, and misinformation can mislead users, especially those with low health literacy or digital literacy skills [6,15]. Critically, disparities in internet access and use, particularly among rural, older, and low-income populations, threaten to widen existing health inequalities rather than close them. Consequently, promoting digital health literacy (e.g., the ability to find, understand, evaluate, and use online health information) is increasingly recognized as a vital component of public health strategies to ensure that online resources improve rather than hinder health outcomes.

The COVID-19 pandemic significantly accelerated the public’s reliance on the internet for health information, services, and care delivery. While trends already showed steady increases in online health information-seeking behavior, the pandemic made digital access not just convenient, but essential for many [3]. Lockdowns, overwhelmed health systems, and public health guidelines promoting social distancing led to a rapid shift toward telehealth, remote monitoring, and online health education. However, this shift was not experienced equally. Research by Suh and colleagues [16] found that during the pandemic, lower-income and minority communities in the U.S. experienced smaller increases in online engagement with critical resources like health information and unemployment services, highlighting significant digital inequalities tied to socioeconomic and environmental factors. These disparities risk worsening long-term health, educational, and economic outcomes for already disadvantaged populations. Today, even as in-person services resume, many patients and caregivers continue to rely on digital platforms for healthcare access, particularly for mental health services, chronic disease management, and follow-up care. This ongoing digital shift underscores the persistent need for equitable broadband access, technology training, and strong efforts to counter health misinformation, especially for rural, older, and underserved populations.

Indeed, while digital technology has become essential across many life domains, rural populations in the U.S. continue to face persistent challenges in both digital access (infrastructure availability) and digital usage (skills and adoption), contributing to widening inequalities. Many rural areas still lack reliable broadband internet; as of 2023, about 17% of rural Americans lacked access to meeting the FCC’s minimum speed benchmark, compared to just 1% of urban residents, often due to geographic isolation, lower population density, and the high costs deterring investment [10]. Even when broadband is available, affordability remains a significant barrier, with rural residents often paying higher prices for slower services compared to their urban counterparts [3].

Beyond infrastructure and cost, barriers related to usage and adoption further complicate the picture. Rural populations, particularly older adults, individuals with lower educational attainment, and lower-income households, often report lower levels of digital skills, limiting their ability to effectively use online health portals, telehealth, or other digital tools [15]. Disparities also exist in device ownership, with rural residents less likely to own smartphones, tablets, or computers, further reducing opportunities for digital engagement [3]. Additionally, factors like skepticism toward digital technology, concerns about privacy, and a preference for in-person services can contribute to lower adoption rates in some rural areas [17]. While federal initiatives like BEAD and RDOF aim to expand rural broadband, implementation is often slow and uneven, particularly in the most remote or economically depressed regions. Addressing these multifaceted challenges effectively requires comprehensive strategies that go beyond simply building infrastructure, incorporating investments in affordable access, robust digital literacy training, device access programs, and the tailoring of digital services to meet the specific needs and cultural contexts of rural communities. Given these multifaceted challenges and the increasing importance of digital tools in healthcare, there is a critical need to better understand the specific factors influencing how caregivers engage with online health information, particularly in underserved settings.

The challenges of the digital divide are part of a multi-layered context. More broadly framed public health models, such as the Socioecological Framework, state that behavior is shaped by factors across the individual, interpersonal, community, and public health levels [18]. Similarly, health literacy models such as Nutbeam’s hierarchy [19] distinguish between basic literacy (e.g., reading skills), interactive literacy (e.g., communicating with one’s doctor), and critical literacy (e.g., advocating for health resources to be incorporated into one’s community). While these frameworks help to provide an overview of the problem, our study utilizes the more specific lenses of Digital Divide Theory and the Theory of Planned Behavior to examine the specific mechanisms by which individual and structural factors interact to produce information inequities within a community.

While the importance of health information for caregivers is clear, less understood is how specific factors like education and geographic isolation uniquely shape information seeking in underserved rural areas. Therefore, this study focuses on caregivers for older adults in Mississippi to examine how educational attainment and rurality combine to influence their access to, attitudes toward, and engagement with various health information resources.

### 1.3. Literature Review

#### 1.3.1. Rural–Urban Disparities in Health Information Access

Having access to accurate and timely health information is crucial for both individual and community health outcomes. However, persistent rural–urban disparities in health information access contribute significantly to broader health inequities in the United States [20]. Rural areas face severe healthcare provider shortages compared to urban areas. According to the Health Resources and Services Administration [9], more than 60% of federally designated HPSAs are in rural locations. Rural counties have fewer physicians per 100,000 residents, with some areas lacking specialists altogether, leading to delayed diagnoses and treatments [21]. Furthermore, rural hospital closures have exacerbated healthcare access issues. Between 2010 and 2023, over 140 rural hospitals closed in the United States, with the South (including Mississippi) being the most affected region [22]. This trend has forced rural residents to travel greater distances for emergency care, preventive services, and specialist consultations.

Beyond physical healthcare access, rural residents often face limitations in accessing health information itself. According to Chen and colleagues [23], rural residents report inadequate access to health information from both in-person sources (like primary care providers and specialists) and online sources (such as search engines, blogs, and magazines). Empirical evidence thus clearly shows that rural residents often have fewer reliable information channels compared to urban residents, compounding the challenges posed by fewer physical access points (hospitals, clinics). This combined access barrier contributes to poorer health outcomes and greater health disparities.

Compounding these healthcare and information access issues, the well-documented ‘digital divide’ further restricts pathways to health resources [24]. While infrastructure availability remains a challenge [10], broadband adoption (subscribing to and using available services) presents a distinct, equally important barrier. Rural adoption rates consistently lag urban areas, often hindered by factors including affordability, lack of perceived relevance, and lower average levels of digital literacy [10,15]. Furthermore, studies indicate that rural populations often exhibit lower average health literacy levels, which can complicate their ability to seek, understand, and apply health information obtained from any source, whether traditional or digital [25]. This combination of lower digital and health literacy, often linked to demographic factors like educational attainment, age, and income prevalent in some rural areas [15], significantly limits the ability to effectively search for, evaluate, and use digital health information. A recent systematic review confirmed that rural individuals in the USA face a variety of socioeconomic barriers, including lower income and educational levels, that together exacerbate the health divide between them and their urban counterparts [26].

Consequently, low broadband adoption and limited digital and health literacy reduce the capacity of many rural residents to fully engage with beneficial resources like telehealth, online health information portals, and other digital tools [17]. These disparities contribute not only to health inequities but also to broader social and economic disadvantages compared to urban populations. Addressing these gaps requires comprehensive approaches; even as broadband infrastructure expands through programs like BEAD, parallel investments in affordable service options and community-based digital and health literacy training are essential to ensure meaningful adoption and beneficial outcomes.

#### 1.3.2. The Digital Divide Theory (DDT)

Following Wei and Hindman’s Digital Divide Theory (DDT) framework [27], we can distinguish between the access divide (infrastructure), use divide (behavioral adoption), and outcome divide (benefits gained). This aligns with Van Dijk’s [28] emphasis on material, skills, and usage access.

Access Divide: The access divide refers to inequalities in the physical infrastructure and hardware required to connect to digital technologies. This dimension represents the most basic level of the digital divide, focusing on whether individuals can physically access devices and internet connectivity. Research by Whitacre and colleagues [17] provides concrete evidence of this divide, showing that healthcare facilities in nonmetropolitan counties connect with significantly lower speeds than their urban counterparts. More concerning, this connectivity gap grew during 2010–2014, with over 55% of metropolitan institutions having download speeds exceeding 50 MBPS by 2014, compared to only 12% of nonmetropolitan institutions [17].

Use Divide: The use divide represents a more nuanced understanding of digital inequality, focusing on how people engage with technology once they have access. This dimension blends structural and behavioral components, including digital skills, literacy, and patterns of engagement. Van Dijk [28] elaborates on this by identifying multiple layers of access beyond mere physical connection: (a) Motivational access (willingness to use technology); (b) Skills access (operational, informational, and strategic digital skills); and (c) Usage access (frequency, diversity, and sophistication of use). The use divide emphasizes that having technical access does not automatically translate to meaningful engagement. Wei and Hindman [27] found that socioeconomic status (especially education) was significantly more associated with informational internet use than with internet access, highlighting that how people use technology is often more unequal than whether they have access to it.

Outcome Divide: The outcome divide represents the ultimate impact of digital technologies on people’s lives: what users can achieve from their engagement with technology. This is where structural, behavioral, and contextual factors collide to determine whether digital engagement translates into tangible benefits. Wei and Hindman found that the knowledge gap (e.g., differences in political knowledge by socioeconomic status) was wider among internet users than among users of traditional media [27]. This suggests that digital technologies may actually amplify existing inequalities in outcomes rather than reducing them.

#### 1.3.3. Rural Populations and the Triple Divide

Rural populations often experience all three types of digital divide simultaneously, creating compounded disadvantages:

1. Access challenges: Whitacre and colleagues [17] documented a persistent and growing connectivity gap for rural healthcare facilities. By 2014, the gap in high-speed connectivity (>50 MBPS) between urban and rural healthcare institutions had widened significantly.
2. Use barriers: Chen and colleagues [23] found that rural residents had lower access to several health information sources. More critically, rural residents with limited health literacy had significantly lower access to mass media and scientific sources but were more likely to use commercial company sources, which may not always provide reliable health information.
3. Outcome disparities: Jongebloed and colleagues [29] identified multiple barriers to effective digital health technology use in rural communities, including product complexity, reliability issues, awareness gaps, trust concerns, and cost factors. These barriers can prevent rural residents from realizing the potential benefits of digital health technologies even when they have access.

Ji and colleagues [30] further demonstrated that both digital literacy and health literacy impact rural residents’ participation and diversity in digital health behaviors, with these factors having a substitutive interaction effect. This suggests that improving both types of literacy is essential for enhancing rural residents’ health outcomes and bridging the urban-rural health divide.

Taken together, prior studies indicate that the evolution of digital divide research has moved beyond simple binary notions of access versus no access toward a more nuanced understanding of how multiple, recursive layers of inequality interact. As Van Dijk [28] argues, the digital divide consists of interdependent types of access, with inequalities in skills and usage becoming increasingly critical. For rural populations specifically, addressing the digital divide requires comprehensive approaches that target not just infrastructure limitations but also skills development and meaningful engagement. As demonstrated by studies across different rural contexts (United States, Australia, and China) [15,20,29,30,31], the challenges are both structural and behavioral, requiring interventions at multiple levels to ensure that digital technologies serve as tools for inclusion rather than mechanisms that reinforce existing disparities.

#### 1.3.4. The Theory of Planned Behavior (TPB)

The Theory of Planned Behavior (TPB) offers a valuable framework for predicting and explaining human behavior within specific contexts, bridging the gap between purely physiological or broadly institutional explanations of action [32]. At its core, TPB, an extension of the earlier reasoned action theory, suggests that behavioral achievement is derived from both motivation and ability. Motivation, in this sense, is influenced by an individual’s assessment of resources, opportunities, and the likely outcomes of a performed behavior. Ability encompasses not only the possession of necessary skills but also the perception of behavioral control (e.g., the perceived ease or difficulty of performing the behavior in question) [32]. Accordingly, TPB posits that behavior is driven by behavioral intentions, which are themselves shaped by three primary constructs: attitudes toward the behavior, subjective norms, and perceived behavioral control [32,33].

The relevance of TPB to health information-seeking behaviors has become particularly evident as individuals increasingly turn to online resources. The COVID-19 pandemic, for instance, significantly accelerated the use of online platforms for health-related information and spurred the adoption of telehealth services, a trend that continues to shape healthcare interactions. In this evolving landscape, understanding the factors that drive engagement with digital health information is critical. Notably, recent research suggests that, contrary to some earlier assumptions about digital skepticism, rural caregivers may hold more favorable attitudes toward online health information than previously thought [33]. Indeed, individuals’ attitudes, particularly their trust in the specialized information available online, can be a powerful motivator for seeking such information. This underscores the importance of trustworthy and accessible digital health resources, especially for populations managing complex caregiving responsibilities.

Each of Ajzen’s concepts of planned behavior is itself relevant to the seeking of health information by caregivers. For example, attitudes toward behavior may take the form of a belief in the degree to which health information one comes across is trustworthy and beneficial. In this study, we conceptualize ‘attitude’ as a multidimensional evaluation of an information source, based on beliefs about its specific attributes, including its trustworthiness, accuracy, clarity, timeliness, and overall usefulness.

Additionally, as found by Chow and colleagues, the perceived trustworthiness of the information shapes the degree to which caregivers will engage with digital health platforms [34]. The second component of TPB, subjective norms, can take the form of caregivers’ perceived expectations of how their family or friends regard particular health behavior decisions, and can, in turn, impact their use. Finally, the last aspect of TPB, perceived behavioral control, can be expressed within our study’s parameters as the self-assessed degree of difficulty in obtaining needed health information. This may relate to digital literacy or access challenges and can play a more significant role when digital tools are seen as relevant [35]. It is important to note that education level may shape both the attitudinal and perceived behavioral control components of TPB, potentially affecting both intention and behavior of our sample.

#### 1.3.5. Education as a Moderator

Education typically enhances attitudes and perceived control by increasing digital health literacy [36]. In their study of 4974 American adults, Mackert and colleagues found that health literacy was positively associated with perceived ease of use and usefulness of health information technology (HIT) tools. Those with higher health literacy were significantly more likely to adopt various HIT tools, including fitness apps, nutrition apps, activity trackers, and patient portals, suggesting that education facilitates digital health engagement by enhancing users’ ability to navigate and derive value from these technologies.

However, Chow and colleagues [34] found that more educated caregivers were more concerned about data privacy, which could moderate their attitudes toward digital health sources. Their mixed-methods study of 1128 caregivers revealed that urban residents and those with higher education levels exhibited greater concerns about online privacy compared to rural residents and individuals with lower educational attainment. This suggests that education may simultaneously enhance capacity for digital engagement while also increasing awareness of potential risks, creating a nuanced relationship between education and digital health tool adoption.

Context-Dependent Associations: This relation is context-dependent. In rural settings, there may be barriers that reduce the effectiveness of education in promoting digital engagement. Viswanath and Kreuter [37] highlighted that disparities in communication technology access remain significant, with higher-income and better-educated groups benefiting more from advancements in eHealth. Importantly, these researchers noted that the usability and cultural relevance of online health information further restrict access for disadvantaged groups, suggesting that education alone cannot overcome structural barriers to digital health engagement in rural areas.

Link and colleagues [35] found that attitude was the strongest predictor of online health information-seeking behaviors, but perceived control is crucial in areas with disparities in digital access. Their study of 822 German internet users revealed that attitudes toward seeking health information online provided the strongest explanatory power for online health information-seeking intention. However, for individuals facing health threats, self-efficacy (a component of perceived control) played a more significant role, suggesting that in contexts where digital access is challenging, confidence in one’s ability to effectively use available resources becomes particularly important.

Rural-Specific Considerations: Park and colleagues [38] further illuminate this dynamic in their mixed-method study of older Korean adults in rural areas. They found that external controls, attitudinal beliefs, and cognitive health had significant positive paths to internal abilities like self-efficacy and skillfulness. While higher education was linked to stronger attitudinal beliefs, the researchers emphasized that external resources and tailored education programs are essential for fostering positive attitudes and reducing barriers to technology use in rural settings. This suggests that standard educational approaches may be insufficient without contextual adaptation.

Digital Divides in Health Information Access: Neter and Brainin [39] provide additional insight into how education influences digital health engagement through their study of eHealth literacy in Israel. They found that respondents with higher eHealth literacy, who tended to be more educated, used a broader range of search strategies and evaluated online health information more critically. Their research suggests that eHealth literacy reinforces existing social inequalities, as individuals with greater resources and education benefit the most from online health information.

In conclusion, the relation between education and digital health engagement is multifaceted and context-dependent. While education generally enhances digital health literacy and facilitates technology adoption, this relationship is moderated by factors such as privacy concerns, rural–urban disparities in access, and the availability of contextually appropriate resources. These findings underscore the importance of developing targeted interventions that address both individual-level factors (such as education and digital literacy) and structural barriers (such as access and cultural relevance) to promote equitable digital health engagement across diverse populations and settings.

#### 1.3.6. Integrating the Two Theories

**Figure 1:**
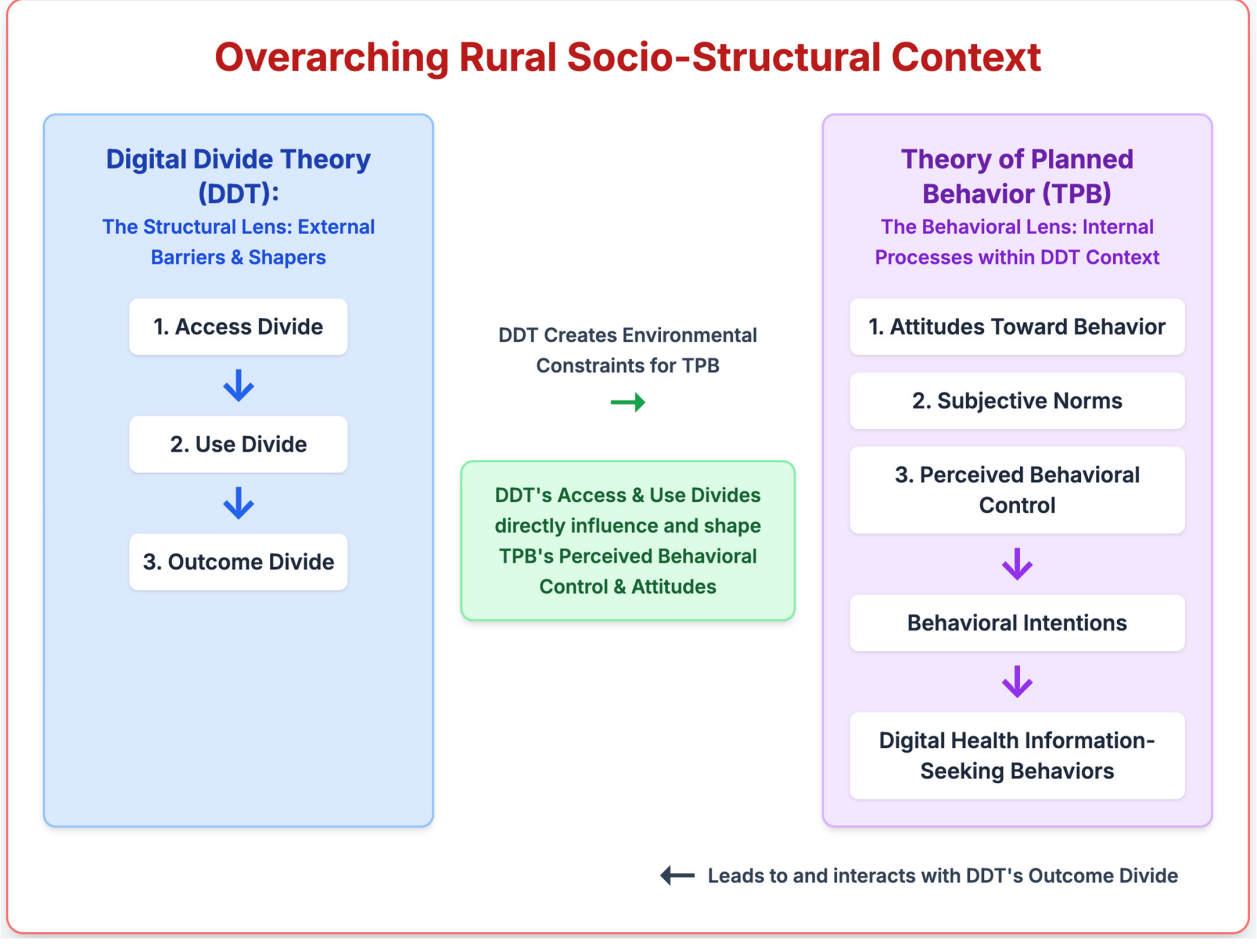
Integrated Conceptual Model Illustrating Digital Divide and Planned Behavior Pathways Influencing Rural Health Information Engagement. Note. This model combines Digital Divide Theory’s structural barriers and the Theory of Planned Behavior’s psychosocial processes to explain caregivers’ digital health behaviors in rural settings.

Our two guiding theories, Digital Divide Theory and the Theory of Planned Behavior, work together to address both the external barriers and internal processes inherent in the digital health information-seeking behaviors of rural older adults. On the one hand, Digital Divide Theory provides a structural lens, by highlighting how the geographic and infrastructural disparities that are common within Mississippi can shape care providers’ access to digital health resources, as well as their use, and potential benefit from these resources. Correspondingly, the Theory of Planned Behavior provides a behavioral lens, by illustrating how the perceptions of these caregivers can influence the efficacy of their information seeking. The external barriers these caregivers face, such as the broadband infrastructure and geographic isolation of rural areas, and the internal processes, such as their levels of trust in online sources and their digital health efficacy, are shaped by this dual-theory framework. Together, the theories help to explain the ways in which rurality may influence both their information-seeking behavior (the structural constraints) and also the ways that they respond to their encountered barriers (as part of their behavioral intentions; see Figure 1).

### 1.4. Current Study

While prior work has established the importance of education and the existence of a rural–urban digital divide independently, limited research has explored their crucial interaction, particularly within structurally underserved contexts like Mississippi. Furthermore, few studies have applied behavioral theories like the Theory of Planned Behavior to explain why digital health engagement differs in these settings.

Therefore, this study aims to address this gap by examining how educational attainment and rurality interact to shape caregiver attitudes toward, and engagement with, digital health information. Drawing on the frameworks of Digital Divide Theory (DDT) and the Theory of Planned Behavior (TPB), we move beyond main effects to test a specific moderation hypothesis. We posit that in a structurally underserved context, the expected positive impact of education on digital engagement may be weakened or even reversed by environmental barriers (e.g., infrastructure quality, cultural norms, affordability), creating a nuanced “outcome divide” not explained by access alone.

### 1.5. Research Questions and Hypotheses

This study investigates how rurality relates to caregivers’ use of, perceptions toward, and difficulties encountered with various health information sources, particularly within the context of Mississippi, a state historically marked by significant disparities in both healthcare access and digital infrastructure. Drawing from the theoretical frameworks of the Digital Divide Theory and the Theory of Planned Behavior (TPB), the following six research questions (RQs), each paired with a corresponding hypothesis (H1–H6), guided the investigation.

- RQ1: Do the primary health information sources that caregivers consult first differ significantly by rurality level?

**H1.**  *Informed by the digital divide’s emphasis on differential access to resources, we hypothesized that caregivers in more rural areas would be less likely than their metropolitan counterparts to consult the internet first, relying instead on friends and family.*

- RQ2: Do caregivers’ attitudes toward health information obtained from (a) the internet and (b) friends and family differ across rurality levels?

**H2.**  *Drawing from DDT, we hypothesized a trade-off in attitudes toward information sources. We predicted that due to the digital divide, caregivers in more rural areas would hold (a) less positive attitudes toward online health information, while a greater reliance on social networks would lead to (b) more positive attitudes toward information from friends and family.*

- RQ3: Does the likelihood that caregivers report difficulty finding needed health information differ based on their level of rurality?

**H3.**  *Drawing from the “outcome divide,” which posits that rural residents face greater structural barriers to successfully acquiring information, we hypothesized that caregivers in more rural areas would report a higher likelihood of difficulty in finding the health information they need.*

- RQ4: Does a caregiver’s level of rurality predict their likelihood of using the internet for health information searches?

**H4.**  *Consistent with the ‘access’ and ‘use’ dimensions of the DDT, we hypothesized that higher rurality would predict a lower likelihood of caregivers using the internet for health information searches.*

- RQ5: Does rurality moderate the relation between a caregiver’s education level and their attitude toward internet-based health information?

**H5.**  *Drawing on our guiding theories* *, which suggest the benefits of individual resources like education are context-dependent (TPB) and can be limited by structural barriers (DDT’s outcome divide* *), we hypothesized that the positive relation between caregiver education and attitudes toward internet health information would weaken as rurality increases.*

- RQ6: Does the perceived accessibility of internet health information mediate the relation between rurality and caregivers’ reported difficulty in finding needed health information?

**H6.**  *Guided by TPB’s pathway model and DDT’s focus on access barriers, we hypothesized an indirect effect. Specifically, we predicted that higher rurality would be associated with lower perceived accessibility of internet health information (Path A), which in turn would predict a higher likelihood of caregivers reporting difficulty in finding that information (Path B).*

Together, these questions not only evaluate differences across rural–urban caregiving contexts but also explore why those differences may emerge, while emphasizing structural and cognitive barriers central to both Digital Divide Theory and the Theory of Planned Behavior.

## 2. Materials and Methods

### 2.1. Participants

Data was collected from a sample of caregivers in Mississippi recruited via CloudResearch panels. The final sample size for analyses varied depending on missing data for specific variables, ranging from N = 249 to N = 452, out of 468 initial responses. Participants’ county of residence was used to assign a 2023 Rural-Urban Continuum Code (RUCC), categorizing them into Metropolitan, Micropolitan, Small Town, or Rural areas (see Section 2.3 for detailed operationalization).

Participants’ ages ranged from 18 to 90 years, with a mean age of 45.90 years (*SD* = 13.45). The majority of participants identified as female, non-Latinx, and Caucasian/White. In terms of education, the most frequent responses were that respondents had completed high school or some college/vocational training. Most commonly, participants were married, employed full-time, and 35% earned less than $25k annually. Full participant demographic characteristics are outlined in Table 1.

### 2.2. Procedure

This study utilized a non-probability, purposive sampling method to recruit participants through CloudResearch online research panels and the Wolfgang Frese Survey Research Laboratory. The study drew upon the Prime Panels participant pool, a resource encompassing millions of individuals from widely used opt-in market research panels, which allows for targeted recruitment based on specific study criteria [40]. The methods for recruiting these respondents varied by the individual panel supplier and included strategies such as email invitations and listings on a central survey HUB website [41]. Prior to initiating data collection, all study procedures and materials were reviewed and approved by the university’s Institutional Review Board (IRB). To be eligible for participation, individuals had to confirm they were 18 years of age or older, currently residing in Mississippi, and actively providing care for at least one adult aged 50 years or older. Potential participants accessed the survey link via the Qualtrics survey platform and first completed screening questions to verify eligibility. Those meeting the inclusion criteria were then presented with an electronic informed consent form. This form outlined the study’s purpose, the nature of participation (completing an online survey), potential risks and benefits, confidentiality measures, and the voluntary nature of involvement, including the right to withdraw at any time without penalty. Participants indicated their consent electronically before gaining access to the full survey, which was administered online. Upon completion of the survey, participants received compensation through the CloudResearch system according to their agreement with the panel provider [41].

### 2.3. Measures

Data for this study was collected via a statewide survey administered through CloudResearch. The survey assessed caregiver demographics, caregiving context, health information-seeking behaviors, and attitudes toward different information sources. Key variables used in the analyses are described below.

- Rurality. Geographic rurality was operationalized using the 2023 Rural-Urban Continuum Codes (RUCC) provided by the U.S. Department of Agriculture (USDA) Economic Research Service (ERS) [12]. These codes classify all U.S. counties along a 9-point continuum where ‘1’ represents counties in metropolitan areas of 1 million population or more, and ‘9’ represents completely rural counties with less than 2500 urban population and not adjacent to a metro area. For each participant, the 2023 RUCC score corresponding to their reported Mississippi county of residence was assigned. This 9-point RUCC score was used as a continuous variable in regression analyses to capture the full spectrum of rurality, with higher scores indicating greater rurality.
  For descriptive purposes and some analyses requiring categorical comparisons (e.g., for RQ1 and RQ3), this 9-point RUCC score was categorized into four groups based on the USDA ERS definitions and your specified population thresholds:
  ○ Metropolitan: Counties with an RUCC of 1, 2, or 3 (typically representing populations of 250,000 or more).
  ○ Micropolitan: Counties with an RUCC of 4 or 5 (typically representing populations between 20,000 and 249,999).
  ○ Small Town: Counties with an RUCC of 6 or 7 (representing non-metro counties with urban populations of 2500 to 19,999).
  ○ Rural: Counties with an RUCC of 8 or 9 (representing non-metro counties with urban populations of less than 2500).

- Primary Health Information Source. Caregivers were asked, “When searching for health information or services on behalf of the person you care for, which information source are you most likely to go to FIRST?”. Response options included: A health service provider, Traditional media (TV, radio, newspaper), Social Media, The Internet, Friends and family, and some other information source. For some analyses, responses were recoded into a dichotomous variable comparing those who chose the internet first versus those who chose Friends/Family first.
- Attitudes Toward Internet Health Information. A composite score was created to measure positive attitudes toward using the internet for health information. This scale sums responses to several Likert-type items assessing trust, usefulness, accessibility, and intent to use the internet for health information in the future, assessing agreement with statements of, “The information was trustworthy,” “The information was up to date,” “The information was accurate,” “The information was presented in a way that was easy to understand,” “If needed, I will seek information from the internet in the future,” “The Internet offered me guidance I could use,” and “I can get information from the Internet when I need it”). Higher scores indicate more positive attitudes.
  To assess the psychometric properties of this investigator-developed scale, we examined its reliability and validity. An analysis of the 8 items comprising the scale yielded a Cronbach’s Alpha of 0.926 (N = 250), indicating excellent internal consistency. The scale demonstrates strong evidence of content validity, as the items were selected to capture key facets of the attitude construct, including perceived trust, accuracy, usefulness, and clarity of online information. Further, evidence for construct validity was supported by a strong, significant positive correlation between the attitude score and the perceived accessibility of the internet (r = 0.827 and r = 0.731 for metropolitan and non-metropolitan groups, respectively), indicating that those who found the internet more accessible also held more positive attitudes toward it as a health information source.
- Attitudes Toward Friends/Family Health Information. A similar composite score measured positive attitudes toward seeking health information from friends and family. This scale sums responses to parallel Likert-type items as those above provided for Internet, assessing trust, usefulness, and intent regarding information from interpersonal sources. Higher scores indicate more positive attitudes.
  A reliability analysis of the 7 items comprising the Attitudes Toward Friends/Family Health Information scale also showed excellent internal consistency, with a Cronbach’s Alpha of 0.933 (N = 229). The items demonstrate content validity by assessing core attitudinal components such as the perceived trust and usefulness of information obtained from interpersonal sources.
- Education. Caregiver education level was assessed and used as a moderator variable in regression analyses. Response percentages are reported in Section 2.1, above.
- Difficulty Finding Information. Caregivers responded to a question asking, “In the past year, was there any health information or services that you could not find?” with yes or no responses.

### 2.4. Data Analysis

All statistical analyses were conducted using IBM SPSS Statistics (version 29.0.2.0). Moderation and mediation analyses specifically utilized the PROCESS macro version 5.0 beta 2.1 [42] within SPSS. Prior to hypothesis testing, data were screened for outliers and assessed to ensure they met the statistical assumptions required for each analysis. For the primary regression-based analyses (including moderation and mediation), these assumptions included the linearity of relationships, the independence of residuals, homoscedasticity, and the normal distribution of residuals. For chi-square tests, assumptions included the independence of observations and adequate expected cell counts. These conditions were evaluated using diagnostic tests and visual inspection of plots within SPSS. Where a notable violation of an assumption occurred, it is reported with the corresponding result, as was the case for the chi-square analysis for RQ1. Missing data were handled using listwise deletion for multivariate analyses. Key variables, particularly the continuous rurality measure (RUCC score) and education level, were mean-centered before inclusion in interaction or mediation models to aid interpretation. An a priori power analysis was conducted using G*Power 3.1 [43] to determine the required sample size for the primary moderation analysis (RQ5). The analysis was designed to have sufficient power to detect a significant interaction effect between caregiver education and rurality on attitudes toward internet health information. The specific test was a linear multiple regression F-test (Fixed model, R^2^ increase).

Several key assumptions were made to perform this calculation. We set the desired statistical power (1 − β) at the conventional level of 0.80, and the alpha level (α) at 0.05. In the absence of prior studies providing a precise effect size for this specific interaction, we conservatively assumed a medium effect size (f^2^ = 0.15) for the R^2^ increase due to the interaction term, following Cohen’s [44] conventions. conventions. The model specified a total of three predictors (education, rurality, and the education × rurality interaction term), with the number of tested predictors being one (the interaction term). This power analysis is also predicated on the assumption that the collected data would sufficiently meet the broader statistical assumptions of multiple regression, such as the normality and homoscedasticity of residuals.

Based on these parameters, the analysis indicated a minimum required sample size of N = 55. The sample size obtained for this specific analysis (N = 249) substantially exceeded this minimum, indicating that the study was well-powered to detect a medium-sized interaction effect if one existed.

The primary independent variable, rurality (based on 2023 RUCC codes), was operationalized in two ways depending on the analysis: (1) As a continuous variable representing the specific 9-point RUCC score assigned to the participant’s county of residence (ranging from 1 = most metropolitan to 9 = most rural), hereafter referred to as the continuous RUCC score. This was used in regression and moderation analyses (RQ2, RQ4, RQ5, RQ6). (2) As a four-category nominal variable (Metropolitan [RUCC 1–3], Micropolitan [RUCC 4–5], Small Town [RUCC 6–7], and Rural [RUCC 8–9]) for descriptive statistics and group comparisons (e.g., RQ1, RQ3). Descriptive statistics (frequencies, means, standard deviations) were computed for all demographic variables, caregiving characteristics, and key study variables related to information sources, attitudes, and perceived difficulties. The specific analytical approaches planned for each research question were as follows:

- RQ1 (Primary Source Differences): To test H1, a chi-square test of independence was conducted. This analysis compared the frequency distribution of the self-reported primary source of health information across the three RUCC categories. A follow-up chi-square test using the dichotomous rurality variable was run to specifically compare Internet vs. Friends/Family as the primary source between metropolitan and non-metropolitan groups.
- RQ2 (Attitudes toward Sources): To test H2a and H2b, bivariate linear regressions directly tested the hypothesized linear relationships by regressing internet attitudes and friends/family attitudes onto the continuous RUCC score.
- RQ3 (Difficulty Finding Information): To test H3, the primary analysis involved comparing the proportion of caregivers reporting difficulty across the three RUCC categories using a chi-square test of independence. This assessed whether the likelihood of experiencing difficulty differed significantly across these geographic groups.
- RQ4 (Predicting Internet Use and Attitudes): To test H4, a binary logistic regression predicted the dichotomous use of the internet variable.
- RQ5 (Moderation by Rurality): To test H5, a moderation analysis using Hayes’ PROCESS macro (Model 1) was conducted. Caregiver education level served as the independent variable (X), the continuous RUCC score as the moderator (W), and positive attitudes toward internet health information as the dependent variable (Y). The significance of the interaction term (Education × RUCC) was examined. If significant, conditional effects of education on positive attitudes towards the internet as a health information source would be explored at low (16th percentile), moderate (50th percentile), and high (84th percentile) levels of rurality, potentially using the Johnson-Neyman technique to identify the range of the moderator where the effect was significant.
- RQ6 (Mediation via Accessibility): To test H6, a mediation analysis using Hayes’ PROCESS macro (Model 4) was run. The continuous RUCC score served as the independent variable (X), perceived accessibility of internet health information as the mediator (M) and reported difficulty finding needed health information as the dichotomous outcome (Y). Given the binary outcome, logistic regression was used within the PROCESS macro to estimate the path coefficients. The significance of the indirect effect (X → M → Y) was assessed using bootstrapping with 5000 resamples to generate bias-corrected 95% confidence intervals.

## 3. Results

Preliminary analyses involved computing descriptive statistics for the sample and key variables (see Table 1). These analyses confirmed the sample characteristics for multivariate analyses and the distribution across RUCC categories. Descriptive summaries also highlighted caregiver demographics (see Table 2), caregiving tasks, information source usage, and perceived challenges. The following sections detail the results pertinent to each research question and its corresponding hypothesis. Analyses were conducted using IBM SPSS Statistics (v29.0.2.0) and the PROCESS macro (v5.0 beta 2.1), with an alpha level of 0.05.

RQ1: Rurality and Primary Information Source

To test H1, which predicted differences in the primary source consulted first based on rurality, a chi-square test compared the distribution of primary sources across the four RUCC categories. The analysis revealed no statistically significant association between rurality category and the primary source first consulted, χ^2^(15, N = 286) = 18.725, *p* = 0.226. This suggests that the initial choice of information source did not significantly vary across metropolitan, micropolitan, small town, and rural caregivers in this sample. However, this result should be interpreted with caution as the assumption of minimum expected cell counts was violated (15 cells (62.5%) have expected count less than 5. The minimum expected count is 0.53). Consequently, H1 was not supported.

RQ2: Rurality and Attitudes Toward Information Sources

H2a predicted that caregivers in more rural areas would hold fewer positive attitudes toward internet health information, while H2b predicted they would hold more positive attitudes toward information from friends and family. Linear regressions using the continuous RUCC score as the predictor were conducted.

- For attitudes toward internet information, the regression model was statistically significant, *F*(1, 248) = 7.867, *p* = 0.005, *R*^2^ = 0.031. However, the relation was positive, *B* = 0.354, SE = 0.126, β = 0.175, t(248) = 2.805, *p* = 0.005. This indicates that caregivers in more rural areas reported significantly more positive attitudes toward internet information, directly contradicting the hypothesis. Therefore, H2a was not supported.
- For attitudes toward friends and family information, the regression model was also statistically significant, *F*(1, 227) = 9.563, *p* = 0.002, *R*^2^ = 0.040. The relation was positive, as predicted, *B* = 0.393, SE = 0.127, β = 0.201, *t*(227) = 3.092, *p* = 0.002. This indicates that caregivers in more rural areas reported significantly more positive attitudes toward information from friends and family. Thus, H2b was supported.

In summary, Hypothesis 2a was not supported, as although there were significant differences by rurality level, rural caregivers held more, not less, favorable attitudes toward internet sources. Hypothesis 2b was supported, with rural caregivers reporting more positive attitudes toward friends and family as sources of health information.

RQ3: Rurality and Difficulty Finding Information

**Figure 2:**
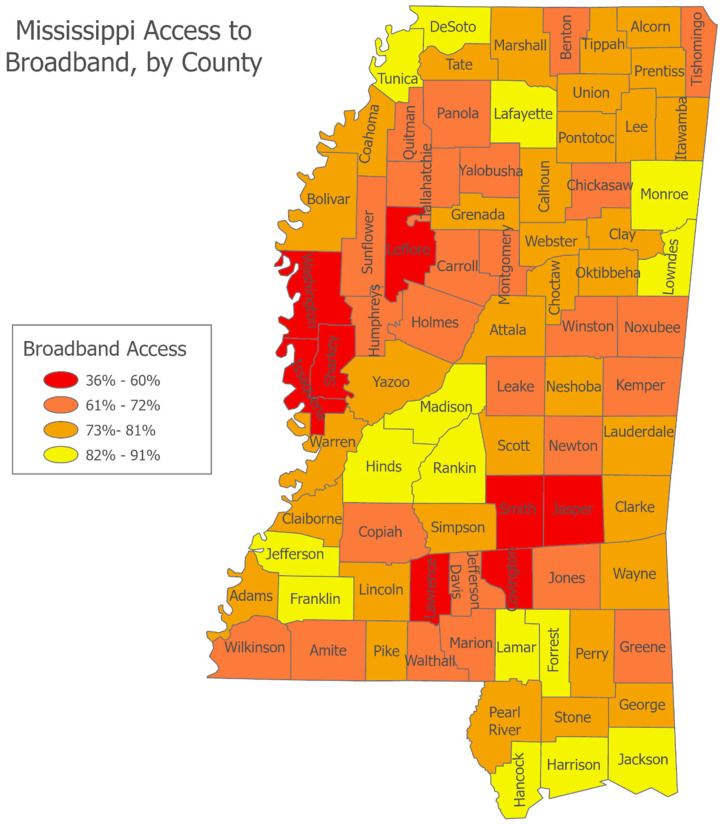
Broadband Internet Access Across Mississippi Counties, 2023. Note. Data provided by the Census Bureau (https://www.census.gov/) and the American Community Survey (https://www.census.gov/acs/www/, accessed on 4 August 2025). Darker shades indicate lower levels of broadband coverage. This map illustrates geographic disparities in digital infrastructure relevant to rural caregivers’ access to online health information.

H3 hypothesized that caregivers in more rural areas would be more likely to report difficulty finding needed health information. A chi-square test comparing reported difficulty across the three RUCC categories found no significant association, χ^2^(1, N = 283) = 1.455, *p* = 0.228 (Cox and Snell R^2^ = 0.005, Nagelkerke R^2^ = 0.007). A binary logistic regression predicting the dichotomous difficulty outcome from the continuous RUCC score also yielded a non-significant result (details under RQ6 results). These findings indicate that the likelihood of reporting difficulty did not significantly differ by rurality level in this sample, B = 0.078, SE = 0.065, Wald(1) = 1.447, *p* = 0.229, Exp(B) = 1.081. (see Figure 2 for broadband access across MS counties). Therefore, H3 was not supported.

RQ4: Rurality Predicting Internet Use

H4 predicted that higher rurality would be associated with a lower likelihood of using the internet for health information. A binary logistic regression predicting use of the internet for health information from the continuous RUCC score found the overall model was not significant, χ^2^(1, N = 285) = 2.100, *p* = 0.147 (Cox and Snell *R*^2^ = 0.007, Nagelkerke *R*^2^ = 0.010). The RUCC predictor itself was not significant, B = 0.088, SE = 0.061, Wald(1) = 2.088, *p* = 0.148, Exp(B) = 1.092. Although not significant, the positive direction of the coefficient (B = 0.088) suggests a slight tendency for those in more rural areas to report using the internet, which is contrary to the hypothesis. However, as the relationship was not statistically significant, rurality did not significantly predict the likelihood of using the internet for health information in this sample. Thus, H4 was not supported.

RQ5: Moderation of Education-Attitude Link by Rurality

H5 predicted that the relations between caregiver education and positive attitudes toward internet health information would be weaker in more rural areas. A moderation analysis using PROCESS Model 1 was conducted with mean-centered variables (*N* = 249). The overall model predicting positive attitudes toward gathering health information via the internet was significant, *F*(3, 245) = 7.0429, *p* = 0.0002, *R*^2^ = 0.0794, using HCO standard errors. There was a significant main effect for RUCC score (*B* = 0.3801, SE = 0.1292, *t* = 2.9412, *p* = 0.0036), but the main effect for education was not significant, *B* = −0.1334, SE = 0.2194, *t* = −0.6082, *p* = 0.5436. Crucially, the interaction term (Education × RUCC) was statistically significant, *B* = −0.2943, SE = 0.0926, *t* = −3.1772, *p* = 0.0017, indicating that the relation between education and attitudes toward internet health information significantly varied depending on the level of rurality.

Simple slopes analyses examining the conditional effect of education at different levels of mean-centered rurality revealed:

- At low rurality (16th percentile, centered RUCC ≈ −2.7621), higher education significantly predicted more positive attitudes (*B* = 0.6803, SE = 0.3029, *t* = 2.2459, *p* = 0.0256).
- At moderate rurality (50th percentile, centered RUCC ≈ 0.4297), the relation was non-significant (*B* = −0.1334, SE = 0.2194, *t* = 0.6082, *p* = 0.5436).
- At high rurality (84th percentile, centered RUCC ≈ 3.7189), the relation significantly reversed; higher education predicted fewer positive attitudes (*B* = −0.9705, SE = 0.3371, *t* = −2.8786, *p* = 0.0044).

**Figure 3:**
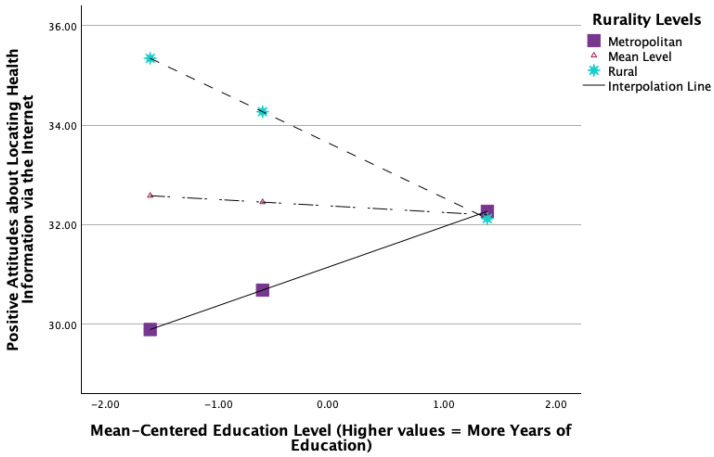
Interaction Between Education and Rurality Predicting Positive Attitudes Toward Internet Health Information. Note. *N* = 249. Simple slopes show that higher education predicts more positive attitudes in low-rurality settings and fewer positive attitudes in high-rurality settings, consistent with an outcome divide.

These findings demonstrate that the effect of education on attitudes toward internet health information diminished and eventually reversed as rurality increased. Therefore, H5 was supported (see Figure 3).

RQ6: Mediation of Rurality-Difficulty Link via Accessibility

H6 hypothesized that the perceived accessibility of online health information would mediate the relation between rurality (continuous RUCC score) and reported difficulty finding desired health information. A mediation analysis using PROCESS Model 4 (*N* = 253, with a dichotomous outcome) was conducted:

- Path a (X → M): Rurality significantly predicted perceived accessibility, *B* = 0.0490, SE = 0.0202, *t*(251) = 2.4285, *p* = 0.0159. The positive coefficient indicated that higher rurality was associated with higher perceived accessibility, contrary to the hypothesized direction.
- Path b (M → Y, controlling for X): Perceived accessibility did not significantly predict the log-odds of reporting difficulty, B = −0.2610, SE = 0.2555, Z = −1.0216, *p* = 0.3070.
- Path c′ (X → Y, controlling for M): The direct effect of rurality on the log-odds of reporting difficulty was also non-significant, B = −0.0866, SE = 0.0909, Z = −0.9527, *p* = 0.3407.
- Indirect Effect (X → M → Y): The bootstrap analysis (5000 resamples) yielded an indirect effect of −0.0055 (BootSE = 0.0133). The 95% bias-corrected confidence interval [−0.0330, 0.0227] included zero, indicating that the indirect effect was not statistically significant.

Given that the non-significant indirect effect and the finding that the X → M path was significant but in the opposite direction of that hypothesized, H6 was not supported.

## 4. Discussion

### 4.1. Summary of Key Findings

Findings suggest that individuals across the rural–urban continuum in Mississippi do not show statistically significant differences in the primary sources they initially consult for aging-related needs. While this result did not meet the threshold for significance at an alpha of 0.05, potential differences warrant further investigation, especially given that low expected cell counts advise a cautious interpretation of these specific results. The lack of differences in this initial information-seeking behavior may be driven by habit or perceived accessibility. Before making policy, public health practice, communications, or marketing decisions in this area, additional data collection efforts and analyses are warranted.

This study also showed that, contrary to hypotheses, caregivers in more rural areas reported significantly more positive attitudes toward online health information. This defies expectations that individuals in rural areas may have less familiarity or comfort with digital resources. Consistent with the hypothesis, caregivers in more rural areas reported significantly more positive attitudes toward information from friends and family. Together, these findings support the notion that rural caregivers value both traditional interpersonal networks and increasingly hold positive views of online resources, challenging simple deficit narratives of many previous studies. This may also reflect a general tendency of individuals in rural areas to be more connected and willing to take on more traditional family caregiving roles.

In examining the difficulty of accessing needed information (RQ3), no significant differences were found across rurality categories, as indicated by the chi-square test results. Additionally, logistic regression analysis revealed that the continuous rurality score did not significantly predict difficulty in finding information (path c′ from RQ6). These findings suggest that factors beyond geographic location, such as the specific type of information needed, digital literacy, health system navigation skills, and the ability to assess information quality, may play a more substantial role in the reported challenges of information access within this sample.

In exploring rurality’s role in predicting internet use for health information (RQ4), the team found that rurality did not significantly influence the likelihood of using the internet for this purpose. This indicates that a simple lack of use or inherent distrust may not fully explain rural caregivers’ engagement with online health information. Interestingly, attitudes towards using the internet for health information are generally positive, even though usage rates are not directly correlated with rurality. It is important to note that our sample consists of individuals who are completing a web survey, indicating that they all have some level of internet access.

The moderation of the education-attitude link by rurality (RQ5) showed that the conditional effect of education on attitudes toward internet information varied by rurality, despite the overall main effect of education being non-significant. Education positively predicted positive attitudes in low-rurality settings, had no significant effect in moderate-rurality settings, and negatively predicted positive attitudes in high-rurality settings. These findings support the notion that the benefits of education for navigating and trusting online health information are context-dependent and may be influenced by factors prevalent in more rural environments, such as infrastructure, the relevance of information to their caregiving situation, and regional digital culture attitudes.

The notion that perceived accessibility to internet service mediates the link between rurality and difficulty finding information was not supported; specifically, the indirect effect was non-significant. Interestingly, while Path a, Rurality → Accessibility, was significant, it was positive, indicating that higher rurality predicted greater perceived accessibility; this is contrary to initial assumptions. However, Path b, Accessibility → Difficulty, was non-significant, meaning that higher perceived accessibility did not translate into lower reported difficulty in finding information. These findings imply that perceived ease of accessing internet information does not explain the differences (or lack thereof) in difficulty finding information. Although rural caregivers perceive online information as accessible, this perception does not alleviate the reported difficulties, suggesting that the bottleneck lies elsewhere. Potential factors could include challenges in finding relevant and trustworthy information, varying levels of digital literacy, or difficulties in applying the information found. Further explanations for this disconnect could be the presence of social desirability bias in survey responses [45], a higher degree of resilience among rural caregivers reporting accessibility in the face of objective challenges [46], and that general familiarity with digital content may not translate into the specific type of skills needed for complex health searches [30].

### 4.2. Theory-Driven Interpretation of Results

Digital Divide Theory: The moderation effect observed in our RQ5 provides compelling evidence for the outcome divide dimension of digital inequality. Education yields significantly different outcomes regarding attitudes towards internet health information depending on rurality, suggesting that structural and contextual factors limit the benefits of education. This finding aligns with Wei and Hindman’s [27] research, which demonstrated that digital technologies may amplify existing inequalities rather than reducing them. The interaction between education and rurality in our study reveals that while education typically enhances digital engagement capacity, rural residents are not gaining the same benefits from their educational attainment. This outcome divide could stem from several factors including poor broadband quality leading to fewer opportunities to apply digital skills, community-level skepticism, or a lack of locally relevant trusted sources [28]. Notably, the non-significant main effect of education further highlights this complexity. Education alone did not predict attitudes toward internet health information; it only mattered in interaction with rurality, demonstrating that rural context is crucial to understanding this relationship.

Furthermore, our findings challenge traditional conceptions of the access divide described in the literature. Previous studies, such as Whitacre and colleagues’ [17] analysis of the National Broadband Map, documented persistent and growing connectivity gaps between urban and rural areas by focusing specifically on the connection speeds of healthcare facilities and institutions rather than individual caregivers. However, in our sample, which by virtue of its online recruitment, necessarily excludes those without any internet access, rural caregivers reported more positive attitudes toward the internet (RQ2a), did not differ significantly in use patterns (RQ4), and reported higher perceived accessibility (RQ6 Path a). This contradicts the expected pattern of the access divide, where rural populations typically experience disadvantages in physical infrastructure and hardware required to connect to digital technologies. This observation may reflect the nature of our online sample, aligning with findings like those of Neter and Brainin [39] which suggests that individuals successfully engaging with online health resources often possess greater eHealth literacy and the underlying resources needed to overcome initial access barriers. Our results also suggest that among rural residents who do have sufficient internet access to participate in online research, the traditional access divide may be diminishing. This aligns with Park and colleagues’ [38] finding that external resources can foster positive attitudes toward technology use in rural settings, potentially indicating that where infrastructure barriers have been overcome, rural residents may develop particularly positive attitudes toward digital health resources.

Conversely, our findings partially support the use divide dimension of digital inequality. In the mediation results for RQ6, rural caregivers perceived digital health information as accessible. Yet, this accessibility did not lead to higher rates of finding necessary health information when needed, underscoring Chow and colleagues’ [34] observation that education has a nuanced relationship with digital health tool adoption. This suggests potential gaps in effective use, possibly reflecting lower digital literacy skills among rural participants, consistent with Ji and colleagues’ [30] finding that both digital and health literacy impact rural residents’ participation in digital health behaviors. However, our rural caregivers already demonstrated unexpectedly strong attitudes toward and access to digital health information (RQ2a and RQ4), challenging aspects of the use divide theory. This nuanced pattern aligns with Chen and colleagues’ [23] finding that rural residents with limited health literacy had significantly lower access to reliable health information sources despite having technical access. Our results suggest that even with access, positive attitudes, and trust, rural caregivers might struggle with finding, navigating, and successfully applying relevant content to their caregiving duties. This finding supports Van Dijk’s [28] assertion that having technical access does not automatically translate to meaningful engagement. Our partial support for the use divide suggests that targeted interventions should focus beyond mere access provision to address skills development and contextual factors, aligning with Jongebloed and colleagues’ [29] identification of multiple barriers to effective digital health technology use in rural communities.

### 4.3. Theory of Planned Behavior (TPB)

While the digital divide framework highlights structural and skills-based disparities, the Theory of Planned Behavior (TPB) offers a complementary lens to understand the individual psychosocial factors (such as attitudes, social influences, and perceived control) that drove caregivers’ engagement with health information in our study. As outlined earlier, within the Theory of Planned Behavior, there are three main components: Attitudes, Subjective Norms, and Perceived Behavioral Control. In line with the TPB, our results supported both the attitudinal (one’s overall evaluation of behavior) and subjective norm (one’s perceptions of social pressure and expectations of those close to you) components. Results from Research Questions RQ2a and RQ2b found that rural caregivers within our sample had more positive attitudes toward obtaining their health information from both internet sources and from informal social network sources like friends and family. The endorsement of health information from friends and family supports the TPB’s subjective norms component, with caregivers being potentially more likely to value these health information sources because it is culturally expected and common in their communities. The endorsement of health information from the internet is support for the attitudinal component of TPB and is different from some previous findings of digital skepticism, which may reflect evolving norms and adapting attitudes toward digital sources within rural communities.

Our results also support the concept of the context dependency aspect of the Theory of Planned Behavior. Our moderation effect from RQ5 shows that the relation between the obtained education level and caregivers’ attitudes towards obtaining their health information from online sources is dependent on the context of their rurality level. In urban settings, higher education levels predicted more positive attitudes toward online health sources, but less positive attitudes in rural settings. These results support TPB’s context dependency framework, because background context is not operating in a vacuum. In this case, the rural context is disrupting the usual pathway between education and the trust level of digital sources.

Finally, our results partially supported the perceived behavioral control (believing that one has the ability to perform an intended behavior) component of TPB. On the one hand, within the results of RQ6 path A, rural caregivers reported higher levels of perceived accessibility, which maps onto this component. However, this did not then translate into better outcomes for these caregivers, and did not predict significant differences in difficulty finding information (RQ6 path B and indirect effects). So, within our sample, having access to the internet did not guarantee successful outcomes in finding needed health information. More research is needed to discover just why this pathway did not translate. Perhaps it is due to gaps in digital literacy, such as trouble identifying which information is trustworthy, or related to challenges in applying information gathered to their own specific caregiving situation. It is crucial to recognize that these individual-level behavioral dynamics, as framed by TPB, unfold within a specific and often challenging broader environment, which the Mississippi context readily illustrates.

### 4.4. Mississippi Context and Structural Considerations

The distinctive context of Mississippi offers an important background for understanding these findings. Despite Mississippi ranking low among the states in access to broadband [47] and healthcare [48], those in our sample from rural areas reported positive attitudes toward gathering their health information via digital means. This finding suggests that where access exists, rural caregivers may be highly motivated to use it, perhaps out of necessity due to a scarcity of in-person services. Concurrently and consistent with regional social norms, our sample reported a strong reliance on gathering health information via interpersonal networks. It is important to consider how these two information-seeking modalities intersect. Rather than viewing them as competing channels, a more nuanced perspective suggests that trust in local informal networks could be used to enhance digital engagement. For example, information from digital sources may be verified or interpreted through conversations with trusted friends and family, and these networks could also spread credible online sources or help build digital skills. Therefore, future interventions should not only aim to provide digital tools but also incorporate them strategically within existing highly trusted social networks that are central to rural communities. Overall, our findings of context-dependent effects of education level on the use and appreciation for digital health services underscore the assertion that interventions must be tailored to our state’s specific rural environments to have the best chance of succeeding, and that one-size-fits-all approaches are likely to fail.

### 4.5. Implications for Public Health and Digital Equity

Our results suggest several avenues for policy recommendations and the creation of clinical interventions. When used to create new interventions targeted toward rural caregivers, our findings indicate that these interventions should leverage the already-positive attitudes toward using the internet to gather health information but should focus on improving effectiveness by enhancing information evaluation skills and usability. This contrasts with prior efforts that just promoted basic access. It is not enough to merely have WIFI or broadband available, nor to try to change negative attitudes, as these were not found to be prevalent in our sample.

Recent studies highlight the importance of developing specific skills, not just gaining access. Broad reviews reveal that digital tools can play a significant role in reducing healthcare disparities in rural areas, but persistent barriers like digital literacy and connectivity issues must be addressed to ensure equity [49]. More targeted systematic reviews of digital health interventions show that structured programs can successfully enhance digital health literacy and improve self-care behaviors among rural and marginalized populations [26,50]. However, these interventions do not always directly translate to improved clinical outcomes. This highlights the same “Outcome Divide” observed in our findings and underscores the complexity involved in translating skills into tangible health benefits [26]. Further, recent empirical work in rural populations confirms that digital literacy and health literacy play distinct but complementary roles. Digital skills may drive caregivers’ initial interactions with online tools, but health literacy is crucial for engaging with a broader variety of health topics [30]. This finding supports our conclusion that effective interventions need to be multifaceted, addressing not only the tools accessed but also the specific skills required to use them effectively.

However, our results do suggest that there are structural barriers present in our population, and these take the form of education levels within rural settings. Our moderation effects indicated that in more resource-limited rural sections of the state, possessing lower education levels did hamper digital health literacy. In these areas, interventions could also utilize trusted interpersonal networks (e.g., by engaging community leaders as digital health ambassadors to champion the initiative) to help disseminate health information and build digital health literacy skills. To help explore why higher perceived accessibility was not reported by our sample to reduce the reported difficulty of finding needed health information, future studies and interventions should also focus on building relevance, trustworthiness, and clarifying the application of the health information conveyed. It is further recommended that policies be developed that help to ensure the continued investment in quality broadband and digital navigation support in rural areas and go beyond mere access by developing health communication strategies that integrate online resources with trusted local support networks. Additionally, policies such as these should support the evaluation of interventions by using clear success metrics, such as improvements in digital health literacy scores and the adoption of verification behaviors (e.g., cross-referencing online information with a provider), to ensure that they are both effective and scalable.

### 4.6. Limitations and Directions for Future Research

Several limitations must be considered when interpreting this study’s findings. First, the reliance on an internet-based survey for recruitment inherently excludes caregivers who do not have internet access. This sampling method may have led to an underestimation of the challenges faced by the most digitally disconnected individuals. However, online surveys are a widely used methodology for collecting data from geographically dispersed populations [51], and this approach allowed us to specifically capture the experiences of rural caregivers who are online. This is a growing group whose positive attitudes toward digital sources within our sample challenged simple Access Divide assumptions.

Secondly, the study’s methodological approach has limitations that also point to promising directions for future research. The cross-sectional design, while useful for providing a snapshot of associations [52,53], prevents us from establishing causal relationships. Likewise, relying on self-report measures, though common for capturing subjective experiences [32], could introduce recall or social desirability biases. To build on these findings and overcome these limitations, future research should use longitudinal designs to monitor digital skills and changing information-seeking behaviors. Including objective measures of digital literacy or behavioral tracking would help clarify causal relationships and offer more practical insights into the specific skills that influence the mechanisms of successful information gathering.

Third, our focus on Mississippi caregivers limits the direct generalizability of findings to other states and regions. For instance, the specific interaction of cultural norms, broadband policies, and healthcare infrastructure found in the Mississippi Delta may differ significantly from the challenges faced by caregivers in other rural contexts, such as geographically isolated communities in West Virginia. However, this geographic specificity also enhances contextual validity by providing deeper insights into how factors within this state, characterized by significant structural inequities, influence outcomes. This insight is crucial for developing regionally tailored interventions. While this focus offers contextual depth, it is also necessary to consider how well the sample represents the Mississippi caregiver population. The sample aligns well with the state’s geographic distribution but appears to underrepresent Black and African American residents and likely overrepresents individuals with higher educational attainment than the state average. Therefore, our findings should be viewed as exploratory and may not be representative of all caregivers across Mississippi, especially those with lower educational attainment and those who lack internet access.

Caution is also advised regarding the chi-square analysis for RQ1, as low expected cell counts reduce the reliability of that particular finding. However, our overall sample size offered enough statistical power for the main regression and moderation analyses at the core of the study.

These limitations, along with the unexpected findings of this study, point toward several promising directions for future research. Methodologically, future studies should strive to include non-internet users by employing mixed-mode data collection strategies, such as integrating paper-based surveys, telephone interviews, or in-person outreach, to gain a more comprehensive understanding of digital health engagement across the entire spectrum of connectivity. Furthermore, given that attitudes and behaviors may shift over time, particularly as digital infrastructure evolves, longitudinal studies are warranted to track the development and persistence of digital health information-seeking patterns among rural caregivers.

Substantively, the quantitative findings point to a need for more targeted research. While this study advocates for enhancing digital health literacy, it did not evaluate its specific components, which is an essential area for future research. To go beyond general recommendations, studies should identify which particular skills are most difficult for rural caregivers. For instance, are they mainly struggling with assessing the credibility of sources, understanding complex medical terms, or applying information in caregiving decisions? Using validated tools like the eHealth Literacy Scale (eHEALS), along with in-depth qualitative interviews, could clarify these specific obstacles. This detailed understanding is vital for creating effective, targeted interventions. Likewise, qualitative research could help to explain the unexpected results showing that rural respondents expressed high trust in online information, and why the advantages of higher education were diminished in more rural areas.

Future research should focus on a more granular analysis, which is a key limitation of this study. Currently, “difficulty” is viewed as a broad idea, but different health topics naturally come with unique challenges. For example, finding mental health information might involve overcoming stigma or evaluating the trustworthiness of advice on sensitive issues, while seeking information about chronic diseases could be complicated by complex medical jargon or difficulties in applying practical instructions. A content-specific approach is needed to identify these subtle barriers and develop more targeted, effective interventions. Additionally, understanding the diversity within rural areas is important. Future research should aim to disaggregate rural communities to explore how local economic and social factors influence digital health-seeking behaviors. Finally, embracing Participatory Action Research (PAR) principles by involving older adults and caregivers (particularly those most affected by digital health disparities) throughout the research process holds significant promise. Such partnerships, grounded in the idea of “nothing about us without us” [54], can enhance the relevance, trustworthiness, and translational impact of research findings, especially in communities where trust in external institutions may be limited.

## 5. Conclusions

This study examined how rural settings, education levels, and caregivers’ engagement with health information interact in Mississippi, using Digital Divide Theory and the Theory of Planned Behavior. Our results challenge simple views of rural digital exclusion. While prior research often emphasizes an “Access Divide,” our findings reveal a persistent “Outcome Divide,” where having internet access does not necessarily lead to real benefits. This is especially clear in the significant moderation effect, showing that the positive influence of higher education on attitudes toward online health info diminishes or even reverses in very rural areas. This indicates that structural barriers can hinder the usefulness of individual resources.

Moreover, our study uncovered an interesting paradox: rural caregivers in our sample viewed online health information as highly accessible, yet this belief did not significantly lessen their reported difficulty in finding the health information they needed. This gap suggests that the main barriers might not be related to internet access but instead involve challenges in effectively navigating, evaluating, and applying online information (i.e., the “Use Divide”).

Finally, these findings provide clear guidance for policymakers and public health practitioners. As digital health initiatives continue to expand, interventions must expand beyond the singular goal of improving infrastructure. To bridge the persistent Outcome and Use Divides, a multi-pronged strategy is necessary. Policies must simultaneously invest in enhancing digital health literacy while also building trust in online health information that is culturally relevant and practical for the needs of caregivers. Acknowledging and addressing this complex interplay of access, skills, and context is critical for developing more effective and equitable digital health support for rural caregivers in Mississippi and beyond.

## Figures and Tables

**Table 1:** Participant Demographic Characteristics.

Characteristic	Categories	*N*	Valid %
Age	18–39	156	34.9
	40–59	205	45.9
	60+	86	19.2
Gender	Female	312	69.3
	Male	137	30.4
	Other (self-identified)	1	0.2
Marital Status	Married	150	33.6
	Unmarried couple	66	14.8
	Divorced	74	16.6
	Widowed	20	4.5
	Separated	28	6.3
	Never married/single	109	24.4
Race	White	301	67.2
	Black/African American	127	28.3
	American Indian/Alaska Native	1	0.2
	Asian	3	0.7
	South Asian	2	0.4
	Middle Eastern/North African	1	0.2
	Multiracial	9	2.0
	Other	4	0.9
Ethnicity	Hispanic or Latino	12	2.7
	Not Hispanic or Latino	435	97.3
Education	Less than high school	21	4.7
	High school or GED	120	26.8
	Some college/vocational	127	28.3
	Associate degree	67	15.0
	Bachelor’s degree	67	15.0
	Master’s degree	35	7.8
	Beyond master’s	11	2.5
Employment Status	Employed full-time	192	42.6
	Employed part-time	42	9.3
	Self-employed	56	12.4
	Out of work >1 year	28	6.2
	Out of work <1 year	18	4.0
	Student	8	1.8
	Retired	41	9.1
	Unable to work	32	7.1
	Choose not to work	7	1.6
	Other	27	6.0
Annual Household Income	<$10,000	30	6.8
	$10K–$14,999	39	8.8
	$15K–$19,999	44	9.9
	$20K–$24,999	42	9.5
	$25K–$34,999	49	11.0
	$35K–$49,999	68	15.3
	$50K–$74,999	85	19.1
	$75K–$99,999	32	7.2
	$100K–$149,999	23	5.2
	$150K–$199,999	16	3.6
	$200K+	13	2.9
	Not sure	3	0.7
RUCC Classification	Metropolitan	206	45.6
	Micropolitan	92	20.4
	Small Town	63	13.9
	Rural	91	20.1

Note. Percentages reflect valid responses only and may not sum to 100% due to rounding. Some categories had missing data or participants who chose not to answer. *N* = 452. Participants are Mississippi caregivers of adults aged 50+.

**Table 2:** Zero-Order Correlations Among Key Study Variables for Metropolitan and Non-Metropolitan Caregivers.

Variable	1	2	3	4	5	6	7	8
1. Positive Attitudes: Internet	—	0.537 **	b.	0.827 **	0.011	–0.014	0.113	–0.012
2. Positive Attitudes: Friends/Fam	0.493 **	—	0.117	0.370 **	0.074	0.022	0.035	–0.083
3. Internet Use	b.	0.029	—	b.	−0.015	0.002	−0.070	0.097
4. Internet Accessibility	0.731 **	0.399 **	b.	—	–0.037	−0.011	0.137	–0.076
5. Difficulty Finding Health Info	0.016	–0.131	−0.017	–0.047	—	−0.043	0.024	−0.017
6. Rurality Level	0.310 **	0.345 **	−0.021	0.304 **	–0.211 **	—	0.019	0.027
7. Education Level	–0.131	–0.242 **	−0.006	–0.067	0.268 **	–0.144 *	—	0.146 *
8. Participant Age	0.170 *	0.274 **	0.171 *	0.127	0.088	0.211 **	0.230 **	—

Note: *N* ranges from 88 to 270. * = *p* < 0.05, ** = *p* < 0.01. Metropolitan caregivers are above the diagonal, and non-metropolitan caregivers are below. Dichotomous variables utilize Point-Biserial correlations. b. = cannot be computed because at least one variable is constant.

## Data Availability

The data supporting the findings of this study are available from the corresponding author upon reasonable request. Data are not publicly available due to participant confidentiality and institutional data-sharing policies.

## Abbreviations

The following abbreviations are used in this manuscript:

ACL	Administration for Community Living
BEAD	Broadband Equity, Access, and Deployment
BEAM	Mississippi Broadband Office
DDT	Digital Divide Theory
EHR	Electronic Health Record
FCC	Federal Communications Commission
HHS	U.S. Department of Health and Human Services
HIT	Health Information Technology
HPSAs	Health Professional Shortage Areas
IRB	Institutional Review Board
PAR	Participatory Action Research
RDOF	Rural Digital Opportunity Fund
RQ	Research Question
RUCC	Rural–Urban Continuum Area
TPB	Theory of Planned Behavior
USDA	United States Department of Agriculture
